# Commentary: IL-17 in Chronic Inflammation: From Discovery to Targeting

**DOI:** 10.3389/fphar.2016.00250

**Published:** 2016-08-11

**Authors:** Francesco Maione

**Affiliations:** Department of Pharmacy, University of Naples Federico IINaples, Italy

**Keywords:** autoimmune disease, cardiovascular disease, IL-17A, IL-17RA, platelets

A recent article titled “IL-17 in chronic inflammation: From discovery to targeting,” by Beringer et al. (2016), greatly reviewed the biology of interleukin-17 (IL-17) family members highlighting the contributions of IL-17 cytokines toward diseases and IL-17-based treatment options. Authors attractively reported how preclinical and clinical studies have provided a solid scientific justification for targeting IL-17 and/or IL-17 receptor (IL-17RA) in human diseases such as inflammatory and autoimmune disorders. Here, I wish to implement the description of the biological properties of IL-17A on the cardiovascular system, the novel underlying mechanisms on platelets aggregation, and thrombus formation, and to report the contribution of IL-17A/IL-17RA axis in the association of autoimmune diseases with cardiovascular risk.

## Autoimmune diseases and cardiovascular risk

Autoimmune disorders comprise different diseases including various rheumatologic, dermatologic, and gastroenterological illnesses. The distinctive feature is the inability of the immune system to turn off the processes directed against the body after an inflammatory response or to prevent its development (Mammen, 2011). The etiology of these complex disorders is unknown. However, the susceptibility to develop autoimmune diseases is due to genetic factors influencing the biochemical events associated with antigen presentation or the mechanisms involved in the development of tolerance (Wu et al., 2016).

There is now compelling evidence that patients affected by autoimmune diseases have a higher incidence of several cardiovascular diseases (Teixeira et al., 2015). It has been suggested that it may depend on the presence of a mutual network in which systemic inflammation, coagulation, fibrinolysis, tissue remodeling, and angiogenesis play closely related roles. The resulting inflammation and organ damage further amplify autoreactive immune responses, forming a self-sustaining and propagating vicious circle (Alkaabi et al., 2003).

During vascular inflammation, cells of innate and adaptive immunity invade the vessel wall. Among them, there are T cells, and some of which produce IL-17A, a pro-inflammatory cytokine produced mainly by a subset of T helper lymphocytes named T_H_17 (D'Acquisto et al., 2010). Therefore, a more complete understanding of the molecular mechanisms involved in the recruitment and activation of T_H_17 immune response should provide insights into the pathogenesis and treatment of these and possibly other inflammatory-based diseases.

## Insight into the involvement of IL-17A on autoimmune-related cardiovascular diseases

T_H_17 cells are characterized by the production of IL-17A (also called IL-17), IL-17F, and IL-22 cytokines that are assumed to be involved in the attack of extracellular pathogens not effectively handled by either T_H_1 or T_H_2 cells. Starting from the premise that T_H_17 cells produce great quantities of IL-17A, most of the T_H_17-mediated effects are attributed to this cytokine (D'Acquisto et al., 2010). IL-17A is a pro-inflammatory cytokine; it is highly produced in patients with chronic inflammatory diseases such as rheumatoid arthritis, multiple sclerosis, intestinal bowel disease, and psoriasis (Beringer et al., 2016). One common feature of patients suffering from these sustained systemic inflammatory conditions is the development of cardiovascular complications including an increased risk of endothelial cell injury, ischemia/reperfusion damage, platelet hyperactivity, and thromboembolism (Alkaabi et al., 2003).

In a study done in 2009, it has been demonstrated that IL-17A *in vivo* sustains rather than induces inflammation, thus amplifying an inflammatory response induced by a pre-existing damage (Maione et al., 2009). In a scientific parallelism, soon after, it has been reported that this cytokine *per se* is unable to induce a pro-aggregating response on murine and human platelets, whereas it able to sustain and amplify platelet hyper-reactivity (Maione et al., 2011). Contextually, it has been reported, for the first time, the presence of a functional role of IL-17 receptor on murine and human platelets (Maione et al., 2011). In contrast with the restricted localization of IL-17, IL-17RA is ubiquitously expressed in different cells and tissues. Moreover, although the pro-inflammatory function and intracellular signaling pathway of IL-17 are strikingly similar to those of IL-1 and Toll receptors, IL-17RA has no homology with other known receptor sequences, thus making IL-17, homologous proteins, and its viral homolog a novel cytokine family. The ubiquitous expression of the IL-17RA gene and its peculiar sequence strongly suggest the possibility of other, as yet, unknown biological functions for this cytokine (Yao et al., 1995).

Consistent with this report, following studies have reported that the combination of IL-17A and TNF-α induces a pro-inflammatory, pro-coagulant, and pro-thrombotic phenotype in human endothelial cells (Hot et al., 2012, 2013). Recent studies also have highlighted that this cytokine facilitates platelet function through the ERK2 signaling pathway (the main intracellular pathway activated in platelets by a number of strong agonist) in patients with acute coronary syndrome (Zhang et al., 2012) and that neutrophil extracellular traps and IL-17A are associated with the organization of thrombi in acute myocardial infarction (de Boer et al., 2013).

Accordingly, recent evidence has also demonstrated that IL-17A is a mediator of angiogenesis, an essential component of chronic inflammation and tissue remodeling associated with autoimmune disorders, which stimulates vascular endothelial cell migration and modulates the production of a variety of pro-angiogenic factors (Numasaki et al., 2003). The latter findings suggest that inhibition of IL-17A may have therapeutic benefits when applied to angiogenesis-related disorders.

Lastly, but not least important, recent *in vivo* evidence has provided preliminary but significant contributions to understand the pro-thrombotic effect of this cytokine. These results show, for the first time, that IL-17A is synergic with a low FeCl_3_ concentration in inducing carotid thrombus in rats and suggest that the effect is likely related to a downregulation of CD39 vascular expression and hydrolyzing activity (Maione et al., 2014).

## Conclusion

Taken together, all these results support the hypothesis that IL-17A plays a crucial role in the development of chronic inflammation and probably in the hemostatic disorders observed in patients with autoimmune diseases. Nevertheless, what remains to be seen is whether *in vitro* evidence, results from animal models and limited *ex vivo* human studies can contribute to better understanding of the IL-17A/IL-17RA axis biology in the context of platelet functionality/hyperactivity and contribute to develop new therapeutic strategies regarding the effects of IL-17A on autoimmune-related cardiovascular diseases. We will see.

## Author contributions

FM designed the General Commentary, drafted the manuscript, and revised it critically for intellectual content.

### Conflict of interest statement

The author declares that the research was conducted in the absence of any commercial or financial relationships that could be construed as a potential conflict of interest. The reviewer AC and handling Editor declared their shared affiliation, and the handling Editor states that the process nevertheless met the standards of a fair and objective review.
